# Biomechanical effectiveness of controlled ankle motion boots: A systematic review and narrative synthesis

**DOI:** 10.1002/jfa2.12044

**Published:** 2024-07-17

**Authors:** Mason L. Stolycia, David E. Lunn, Will Stanier, Josh Walker, Richard A. Wilkins

**Affiliations:** ^1^ Carnegie School of Sport Leeds Beckett University Leeds UK; ^2^ NIHR Leeds Biomedical Research Centre Leeds Teaching Hospitals NHS Trust Leeds UK; ^3^ Physiotherapy Department Leeds Teaching Hospitals NHS Trust Leeds UK; ^4^ Leeds Institute of Rheumatic and Musculoskeletal Medicine University of Leeds Leeds UK; ^5^ Podiatry Department Leeds Teaching Hospitals NHS Trust Leeds UK

**Keywords:** Achilles tendon, function, orthotic walker, rehabilitation

## Abstract

**Introduction:**

Controlled ankle motion (CAM) boots are a below‐knee orthotic device prescribed for the management of foot and ankle injuries to reduce ankle range of motion (RoM) and offload the foot and ankle whilst allowing continued ambulation during recovery. There is a lack of clarity within the current literature surrounding the biomechanical understanding and effectiveness of CAM boots.

**Aims:**

To summarise the biomechanical effects of CAM boot wear as an orthotic for restricting ankle RoM and offloading the foot.

**Methods:**

A systematic literature review was conducted in accordance with the PRISMA 2020 guidelines. All papers were independently screened by two authors for inclusion. Methodological quality was appraised using Joanna Briggs Critical Appraisal checklists. A narrative synthesis of all eligible papers was produced.

**Results:**

Thirteen studies involving 197 participants (113 male and 84 female) were included. All studies were quasi‐randomised and employed a within‐study design, of which 12 studies included a control group and a range of CAM boots were investigated. CAM boots can be seen to restrict ankle RoM, however, neighboring joints such as the knee and hip do have kinetic and kinematic compensatory alterations. Plantar pressure of the forefoot is effectively redistributed to the hindfoot by CAM boots.

**Conclusion:**

The compensatory mechanisms at the hip and knee joint during CAM boot wear could explain the secondary site pain often reported in patients, specifically at the ipsilateral knee and contralateral hip. Although CAM boots can be used to restrict ankle motion, this review has highlighted a lack of in‐boot kinematic analyses during CAM boot use, where tracking markers are placed on the anatomical structure rather than on the boot, or through video fluoroscopy, urging the need for a more robust methodological approach to achieve this. There is a need for studies to assess the biomechanical alterations caused by CAM boots in populations living with foot and ankle pathologies. Future research, adopting a longitudinal study design, is required to fully understand the effectiveness of CAM boots for rehabilitation.

## 1 INTRODUCTION

A controlled ankle motion (CAM) boot is a below‐knee orthotic device which encapsulates the foot and ankle, as well as the shank depending on the model, often prescribed for the management of foot and ankle injuries [1, 2], including the Achilles tendon rupture and metatarsal fractures. They are also prescribed for other medical conditions such as foot ulceration in patients with diabetes mellitus [3]. The purpose of a CAM boot is to allow patients to continue ambulation whilst protecting and immobilising the foot and ankle, and offloading the foot and ankle complex [2].

Different pathologies are often treated with the use of different “styles” of CAM boots; with “tall” CAM boots better suited to those pathologies requiring greater immobilisation, such as acute Achilles tendon rupture where the foot is fixed during the initial 2 weeks [1, 4], whilst “short” CAM boots are prescribed due to their greater convenience and suitability to pathologies that predominantly require offloading, such as diabetes mellitus for healing of plantar ulcerations [1, 5]. CAM boot designs vary greatly; variation in CAM boot rocker soles, such as the thickness of the sole unit, can alter biomechanical gait characteristics (e.g., joint angles) during walking [6], as a result of the incurred leg length discrepancy (LLD) caused by the thickness on the CAM boot sole unit when used with standard footwear at the contralateral limb. However, there are devices that can be used to offset this LLD, with the use of a shoe leveler or “even‐up” worn beneath the sole of the contralateral limb. It has been shown that counteracting and minimising the LLD can have a positive effect on the pain experienced at the knee and hip [7].

CAM boots can be used to reduce plantar pressure around affected areas of the foot, thus allowing for the healing of soft tissue, such as plantar wounds (e.g., ulcers) caused by peripheral neuropathy [5]. Although frequently used, rigid CAM boots are suggested to be of a sub‐optimal design for the healing of such wounds because the immobilisation that a CAM boot achieves, alongside this reduction in plantar pressure, can have detrimental effects to the healing process for patients with already poor or restricted blood flow to the lower extremities [8]; Increasing the risk of complication associated with stasis and loss of the venous calf pump, such as deep vein thrombosis and pulmonary embolism [9].

CAM boots are often prescribed as an alternative to plaster casts because of their ability to be removed, which allows the wound to be regularly checked and cleaned making them more hygienic [10, 11]. Further to this, Beck et al. [11] illustrated no significant difference in pain between the two treatment options, whilst CAM boots boasted a significantly greater range of motion (RoM) and higher patient satisfaction, likely because of the ability to complete ongoing physical therapy during treatment in a CAM boot [12]. When used in tibial fracture patients, CAM boots allow faster return to weight‐bearing and other daily activities without affecting the healing of the fracture [13]. Furthermore, the earlier return to weight‐bearing allowed by the CAM boot is associated with improved mobility, shorter hospital stays, and an earlier return to work [14].

Where the CAM boot offers versatility in its application of uses, it might not provide optimum care for all pathologies it is currently used for, such as the potential implications for patients with poor blood circulation, as referred to earlier. Furthermore, the period of time the boot is used varies between conditions, with Achilles tendon rupture patients spending up to 10 weeks in a CAM boot [15] compared to diabetic foot ulceration where, typically, patients must wear a CAM boot for 6–8 weeks [16]. This affects other joints, with the alterations to gait caused by CAM boots having potential long‐term consequences on neighboring joints like the hip and knee. Ready et al. [2] suggest that CAM boots cause pain predominantly at the ipsilateral knee, contralateral hip, and lower back, with one‐in‐three patients reporting new or worsened pain 3 months post‐CAM boot wear. The incurred LLD can also cause developments of knee and hip osteoarthritis later in life [2, 17]. Therefore, given the potential secondary site consequences of wearing a CAM boot, it is important to understand the effectiveness of CAM boots when they are implemented. A reduction in ankle RoM likely limits the joint's ability to contribute to overall lower‐limb mechanical work, which is a major contributor during walking [18, 19]. As a result, this reduced work potential must be compensated for by the ipsilateral hip and knee joints, or the contralateral limb, if gait speed is to be maintained. This could suggest the reason for slower preferred walking speeds often observed in CAM boot walking compared to shod condition walking [6, 20]. This compensation by neighboring joints could lead to secondary site injury and pain as reported by Ready et al. [2], which could have subsequent effects and costs for the patients, especially those of an older age who could see an early on‐set, or worsening of osteoarthritis at the knee and hip due to overuse caused by the increase in mechanical demand.

Given the discrepancies of the biomechanical effectiveness of CAM boot wear in the literature, as well as the short and long‐term pain and musculoskeletal injury risk, their efficacy could be questioned if the trade‐off between benefits does not outweigh the potential risks when compared to the alterations caused by alternative methods such as casting. Therefore, the aim of this review was to summarise the biomechanical effects of CAM boot wear as an orthotic for restricting ankle RoM and offloading the foot. The objectives of this review are to identify the specific changes in lower extremity joint kinetics, kinematics, foot pressure, and spatiotemporal parameters of gait. This will develop an understanding of the effect of CAM boot wear on patient function during use.

## 2 METHODS

### 2.1 Protocol

The protocol was registered with PROSPERO (registration number CRD42023453137). Reporting is in accordance with the Preferred Reporting Items for Systematic Reviews and Meta‐Analyses (PRISMA) guidelines [21].

### 2.2 Eligibility criteria

Both quantitative and qualitative studies evaluating the use and effectiveness of CAM boots were included. No conference abstracts were included in this review and limitations were placed to ensure only peer‐reviewed papers were included. Further to this, only publications in the English language were included. Any systematic reviews or meta‐analyses were excluded from the review. There were no other restrictions on the study type.

### 2.3 Population

No exclusions were placed on the age of participants used in studies for this review. Only quasi‐randomised studies of healthy participants were included in this review, with any other study design excluded. This allowed for complete analysis of the biomechanical effects of CAM boots with the removal of patient populations ensuring an understanding of boot mechanics only.

### 2.4 Intervention

All included studies must use a CAM boot, defined here as a below knee orthotic device encompassing the foot and ankle.

### 2.5 Comparators

No specific concurrent comparator was required for study inclusion in this review.

### 2.6 Outcome measures

No restrictions were placed on outcome measures.

### 2.7 Search strategy and study selection

The literature search was conducted on October 23, 2023 in the following databases; SPORTDiscus, MEDLINE, CINAHL Complete, and PubMed. Several searches were conducted combining the keywords “controlled ankle motion” OR “range of motion” AND “boot*” OR “orthotic*”, “fracture” OR “diabetes” OR “Achilles”, “offload*” OR “pressure” OR “biomechanics”, “foot” OR “ankle”. An example of the full search strategy is presented in Additional file S1. Once the search had been completed, all duplicate papers were removed manually using Endnote (version 20.0.0.14672). A hand search was performed on Google Scholar to acquire anymore potential papers. Following this, titles and abstracts were screened by two review authors (M.L.S. and W.S.) to identify all eligible articles. Both reviewers independently evaluated and screened any full papers carried forward after abstract screening.

Any disputes regarding suitable papers were discussed between the two reviewers and then escalated to a third independent reviewer (R.A.W.) where necessary.

### 2.8 Data extraction

The two review authors independently performed data extraction using the Joanna Briggs Institute Critical Appraisal checklist for quasi‐experimental non‐randomised studies standardised data extraction form with the following data been extracted from each article: study characteristics (lead author and year of publication), study design, country, mean and range of age, intervention(s), comparator, outcome measures, and summary of key findings. Any discrepancies that occurred after independent data extraction were reviewed through discussion between the two named authors and resolved through discussion with the third reviewer when necessary.

### 2.9 Assessment of methodological qualities

An independent assessment of risk of bias was performed by the two review authors using the Joanna Briggs Institute Critical Appraisal checklist for quasi‐experimental non‐randomised studies, to provide a versatile and comprehensive evaluation of study designs, aligning with the multifaceted nature of CAM boot research. No study was excluded based on their methodological quality.

### 2.10 Analysis

Following methodological quality assessment, studies, and their data, were grouped according to outcome measures. All data were assigned into the following four groups: kinetics, kinematics, spatiotemporal parameters, and foot pressure.

## 3 RESULTS

### 3.1 Study selection

**FIGURE 1:**
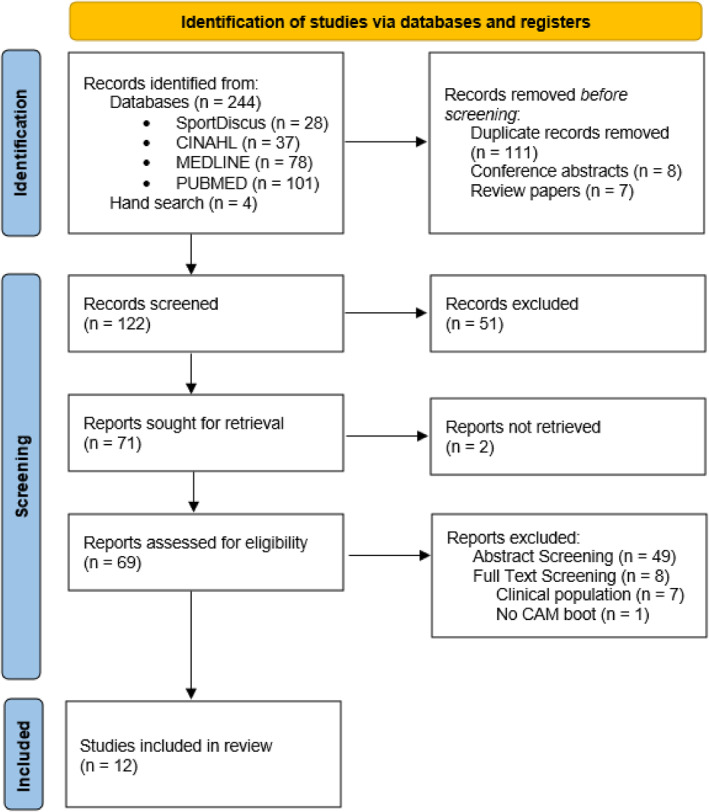
PRISMA 2020 flow chart illustrating the literature search process.

The search yielded 281 articles, of which 13 were retrieved from full‐text screening. All 13 of these studies were in line with the inclusion criteria. The full selection and screening process can be seen in a PRISMA 2020 flow diagram (Figure 1).

### 3.2 Quality assessment of included studies

Methodological quality assessment results are presented in Table 1. All studies were marked “Yes” for six of the nine criteria. All studies were marked as not applicable (‘N/A’) for two of the nine criteria. This was due to studies being a within‐subject experimental trial whereby the data were collected in one testing session, so pre‐ and post‐intervention measures/follow ups were not applicable. Sommer et al. [29] was the only study to be marked “No” for any criteria as it did not have a control group as a comparator.

**TABLE 1:** Methodological quality assessment results.

Study ID	Is it clear in the study what is the “cause” and what is the “effect”?	Were the participants included in any comparisons similar?	Were the participants included in any comparisons receiving similar treatment/care, other than the exposure or intervention of interest?	Was there a control (or comparison) group?	Were there multiple measurements of the outcome both pre and post the intervention/exposure?	Was follow up complete and if not, were differences between groups in terms of their follow up adequately described and analyzed?	Were the outcomes of participants included in any comparisons measured in the same way?	Were outcomes measured in a reliable way?	Was appropriate statistical analysis used?
[22]	Yes	Yes	Yes	Yes	N/A	N/A	Yes	Yes	Yes
[23]	Yes	Yes	Yes	Yes	N/A	N/A	Yes	Yes	Yes
[24]	Yes	Yes	Yes	Yes	N/A	N/A	Yes	Yes	Yes
[25]	Yes	Yes	Yes	Yes	N/A	N/A	Yes	Yes	Yes
[19]	Yes	Yes	Yes	Yes	N/A	N/A	Yes	Yes	Yes
[6]	Yes	Yes	Yes	Yes	N/A	N/A	Yes	Yes	Yes
[1]	Yes	Yes	Yes	Yes	N/A	N/A	Yes	Yes	Yes
[20]	Yes	Yes	Yes	Yes	N/A	N/A	Yes	Yes	Yes
[26]	Yes	Yes	Yes	Yes	N/A	N/A	Yes	Yes	Yes
[27]	Yes	Yes	Yes	Yes	N/A	N/A	Yes	Yes	Yes
[28]	Yes	Yes	Yes	Yes	N/A	N/A	Yes	Yes	Yes
[29]	Yes	Yes	Yes	No	N/A	N/A	Yes	Yes	Yes
[5]	Yes	Yes	Yes	Yes	N/A	N/A	Yes	Yes	Yes

### 3.3 Characteristics of included studies

Table 2 contains an overview of study characteristics. All studies were of a within‐subject study design and did not have any participant follow‐up. Participants were recruited from five different countries: USA, UK, Sweden, Finland, and Switzerland. The sample size of participants recruited for studies ranged from ten [5, 6, 22, 23, 29] to 40 [20], with a total of 197 (113 male and 84 female) participants across all studies. 11 of the 13 studies used a mix of male and female participants, with two using only males [1, 5]. All studies used healthy participants, with seven specifying participants had no previous history of lower extremity injuries [1, 19, 20, 22, 24, 25, 26], two studies making reference to the absence of injury that may affect gait [5, 20], one study made reference specifically to excluding any participant with previous Achilles tendon injury [29], and another to diabetes [23]. Mean participant age ranged from 20.7 to 45.0 years [20, 27], with the mean age reported in all but two of the 13 studies [19, 28]. Age range was reported in four studies [19, 23, 26, 28], ranging from 18 to 59 years.

**TABLE 2:** Overview of study characteristics.

Study ID	Study design	Country	Sample size	Population	Mean age (SD); age range (years)
[22]	Within‐subject experimental	USA	10 M: 6 F: 4	No prior history of lower extremity injury or surgeries	27.2 (±3.6); age range not reported
[23]	Within‐subject experimental	USA	10 M: 5 F: 5	Non‐diabetic	30 (SD not reported); 26–35
[24]	Within‐subject experimental	USA	11 M: 6 F: 5	Healthy participants with no history of major lower extremity injury	27.4 (±7.8); age range not reported
[25]	Within‐subject experimental	UK	15 M: 8 F: 7	Healthy adults (18<) with no previous history of lower limb disorder	31.3 (±4.7); age range not reported
[19]	Within‐subject experimental	USA	20 M: 10 F: 10	Healthy participants with no lower extremity injury 1 year before participation. Never treated for major foot injury. Did not require or use orthotic devices	Mean (SD) not reported; range 19–38
[6]	Within‐subject experimental	UK	10 M: 6 F: 4	Healthy participants	37.1 (±12.1); range not reported
[1]	Within‐subject experimental	USA	14 M: 14 F: 0	No significant injury to the foot and/or ankle and no previous lower extremity surgery	24.1 (±3.5); age range not reported
[20]	Within‐subject experimental	USA	40 M: 20 F: 20	No neurological condition that affected gait, no previous lower extremity surgeries, no lower extremity physical therapy within 6 months	20.7 (±1.8); range not reported
[26]	Within‐subject experimental	USA	20 M: 10 F: 10	Healthy subjects of 18≤ with no history of foot and/or ankle pathology	29 (SD not reported); range 18–59
[27]	Within‐subject experimental	Sweden	16 M: 8 F: 8	Healthy individuals	Male: 45 (±3). Female: 44 (3); ranges not reported
[28]	Within‐subject experimental	Finland	11 M: 5 F: 6	Healthy individuals	Mean and SD not reported; range 26–38
[29]	Within‐subject experimental	Switzerland	10 M: 5 F: 5	Free of acute and/or chronic musculoskeletal, neurological or cardiological diseases	32.5 (±10); range not reported
				No previous issues with Achilles' tendon	
[5]	Within‐subject experimental	USA	10 M: 10 F: 0	Healthy and free from injury and any condition that may alter typical walking patterns	26.6 (±7.5); range not reported

### 3.4 Interventions

All the studies in this review include the use of at least one standard tall CAM boot. Table 3 contains an overview of study interventions and findings. Four studies used only one CAM boot, using different comparator conditions: open and closed toe total contacts casts, and CAM boot with a felt insert [23]; two variations of carbon fiber ankle‐foot orthoses [25]; a post‐operative sandal [19]; and CAM boot with contralateral trainer and CAM boot with contralateral barefoot [20]. Nine studies compared between multiple CAM boots, varying between comparisons of different tall CAM boots [6, 22, 24, 29], tall and short CAM boots [1, 26, 28], and tall and adjustable CAM boots or RoM walker boots [5, 27]. Five of these nine studies use other comparators as well as comparing between different CAM boots: synthetic leg cast [22]; short cast [26]; dorsal brace [27]; post‐operative shoe, forefoot‐relief shoe, and calcaneus fracture orthotic [28]; and novel sprint‐loaded CAM boot [5]. Two studies paired at least one CAM boot condition with the addition of a shoe leveler worn on the contralateral limb [5, 28]. One study completed all intervention conditions with and without crutches [26]. Finally, one paper used the addition of heel wedges with their intervention conditions, creating multiple conditions per intervention [25].

**TABLE 3:** Overview of study interventions and findings.

Study ID	Intervention(s)	Comparator	Outcome measures	Key findings
[22]	Short synthetic leg cast, 4 commercially available short leg walkers: Bledsoe walker boot; Three‐D orthopedic Samson; Royce equalizer; cam walker.	Participant's own shoes	Temporal‐spatial parameters Lower limb joint kinetics and kinematics	Synthetic cast the only condition to significantly alter spatiotemporal parameters. Hip – no significant difference Knee – flexion significantly reduced in cast only Bledsoe the only intervention to report non‐significant changes across all parameters
[23]	Open toe total contact cast; closed toe total contact cast; fracture walker; fracture walker with felt insert	Barefoot	Plantar pressure	All experimental conditions significantly reduced peak plantar pressure at the first metatarsal compared to barefoot. The fracture walker and fracture walker with insert significantly reduced heel pressure
[24]	Two different CAM boots: Gait walker; equalizer	Laboratory running shoes	Ground reaction forces (GRF) and three‐dimensional (3D) joint kinematics and kinetics	CAM boots did not significantly reduce ankle RoM in the sagittal plane Significant increase in ankle sagittal plane moment Significant decrease in ankle eversion Significant increase in knee flexion Significant decrease in hip adduction Both boots increase impact peak GRF Gait walker increases mid‐stance GRF
[25]	Rigid, rocker‐bottom “Aircast walker” CAM boot; carbon‐fiber A ankle‐foot orthosis; carbon‐fiber B ankle‐foot orthosis. All conditions were combined with 3,2,1, and 0 heel wedges all of a 1.5 cm thickness. A total of 4 conditions within each AFO design.	Participants own footwear	Speed; range of motion; heel pressure; forefoot pressure; terminal stance duration	Aircast CAM boot walker showed the greatest reduction in range of movement Reducing the number of inserted heel wedges caused a gradual statistically different decrease in heel pressure as dorsi flexion is a permitted to a greater degree. This caused increased plantar pressure and increased time spent within terminal stance, subsequently.
[19]	Rigid postoperative sandal; CAM boot All tested in 3 gait patterns: Level walking, heel walking, and pivot turns	Standard athletic shoe	Peak pressure; contact pressure; impulse	Short CAM boot significantly reduces peak pressure and contact pressure at the fifth metatarsal in level walking and heel walking when compared to the postoperative sandal, and heel walking relative to the control
[6]	Ossur Rebound Air walker (WA); DJO global Aircast FP walker (WB)	Standardised footwear	Kinetics Kinematics Center of pressure	WA and WB increased knee flexion to SF, WB was significant Significant reductions in knee adduction moments in WA and WB compared to SF WA hip extension moments were significantly different to both WB and SF
[1]	Short CAM boot; tall CAM boot	Barefoot	Sagittal plane talocrural and subtalar joint kinematics	Both CAM boot significantly reduce talocrural RoM, with a significantly greater decrease seen in the tall > short tall boot significantly reduced the subtalar RoM
[20]	Orthopedic boot on right foot, left foot trainer; orthopedic boot right foot, left foot barefoot	Standardised running shoes	Spatial‐temporal parameters Kinematics: Peak joint angles Kinetics: Peak ground reaction force; internal joint moments	Comparing both boot conditions to bilateral shod: significant decrease in walking velocity. All three planes had significant increases in peak pelvic and thorax motion. Significant differences in motion at the knee and hip in all three planes except for the transverse plane on the short limb. Peak ground reaction forces on the long limb were reduced but significant. Peak anterior‐posterior GRF differed significantly across all conditions and side. Hip and knee joint moments were significantly different in all three planes of motion, with the only exception in the frontal plane on the short limb.
[26]	Walking with and without crutches in 3 experimental conditions: Short leg cast, high fracture boot, low fracture boot	Walking with and without crutches in participants own shoes	Sagittal tibiotalar range of motion; peak plantar surface force	Short leg casts and high fracture boots were effective in immobilisation in both weight bearing and non‐weight bearing conditions. Short fracture boots were effective at immobilising the ankle joint during non‐weight bearing only All reduced the peak plantar pressure in comparison to control
[27]	Rigid AFO; adjustable AFO; dorsal brace	Unbraced walking in stockings	EMG activity; plantar pressure; ultrasound of Achilles' tendon displacement	Increasing plantar flexion significantly reduces Achilles displacement in rigid and adjustable AFO. Dorsal brace does not significantly alter displacement Greater plantar flexion progressively decreases forefoot pressure Soleus EMG activity decreases as in‐boot plantar flexion increases. Conversely, tibialis anterior activity increases
[28]	Postoperative shoe; forefoot relief shoe; short walker boot; high walker boot; calcaneus fracture boot; high walker boot with orthotic shoe lift	Running shoes	Peak pressure; contact areas; contact time; force time integral; maximum force	Reduced peak forefoot pressures in all foot orthoses; no significant reductions in midfoot pressures; calcaneus fracture orthosis was the only orthotic to significantly offload the hindfoot
[29]	Three commercially available stability boots from three different manufacturers: Kuenzli; orthotech; OPED	N/A	RoM Joint kinematics Spatiotemporal parameters	Kuenzli and Vacoped have significantly greater plantarflexion immobilisation than orthotech Plantarflexion impulse is significantly different across all three boots and highest in Vacoped Vacoped had significantly lower knee flexion All boots showed between‐limb spatiotemporal differences Between boot center of pressure difference observed
[5]	Traditional CAM boot; range of motion walker boot; custom built, experimental spring‐loaded boot In all boot conditions the boot was worn on the right foot with a shoe leveler worn on the left limb.	Athletic trainers	GRF and plantar pressure metrics; impulse; spatiotemporal parameters; ankle, knee, and hip joint kinematics	All boots significantly reduced ankle range of motion with the traditional CAM boot been the most effective. Spring‐loaded boot showed significantly lower peak pressures in the forefoot and hindfoot when compared to all other conditions.

### 3.5 Kinetic parameters

Kinetic parameters were reported in six of the included studies [5, 6, 20, 22, 24, 29]. Four studies comparing to a control group of bilateral shod illustrated bilateral extensor moments and ipsilateral adductor moments were reduced at the hip and knee [24], with results being significant (*p* < 0.05) in three of these studies [6, 20, 22]. There were no significant changes in knee or hip joint work done during CAM boot wear, although Bruening et al. [5] showed trends toward an increase in both joints.

### 3.6 Kinematic parameters

Nine of the included studies reported kinematic outcome measures [1, 5, 6, 20, 22, 24, 25, 26, 29]. Ipsilateral ankle RoM when wearing a CAM boot was significantly reduced when compared with a control condition [1, 5, 26], although this was not consistent across all papers [24]. Ipsilateral knee flexion increased (*p* < 0.05) [6, 20, 24], whilst contralateral knee flexion decreased (*p* < 0.05) [20]. Ipsilateral knee abduction is significantly reduced, whilst contralateral knee abduction is increased (*p* < 0.05) [20], although not all differences are significant [6, 22]. Hip flexion and adduction were reduced (*p* < 0.05) in the contralateral limb [20] and increased in the ipsilateral limb (*p* < 0.05) [20], although not all findings for hip flexion and adduction were significant [6, 22, 24].

### 3.7 Spatiotemporal parameters

Spatiotemporal parameters were reported in six of the studies included in this review [5, 20, 22, 25, 26, 29]. Trends to suggest CAM boot wear reduces walking speed when compared with control conditions were reported [5, 22], with significant reductions in two studies (*p* < 0.05) [20, 25]. Significant reductions in self‐selected walking speed can be seen in different CAM boots (*p* < 0.05) [29]. Contact time increased significantly in the ipsilateral limb (*p* < 0.05) [20].

### 3.8 Foot pressure

Outcome measures of pressure were reported by six of the included studies [5, 19, 23, 25, 27, 28]. CAM boots significantly decreased forefoot pressure compared to walking in trainers [23, 28] (*p* < 0.05 and *p* < 0.01, respectively) as well as increasing heel pressure (*p* < 0.05) [5, 22, 27], with a correlation between the increase in heel pressure/decrease in forefoot pressure and an increase in plantarflexion through the use of heel wedges [25].

## 4 DISCUSSION

This systematic review aimed to summarise the biomechanical benefits and implications of CAM boot wear as an orthotic device for restricting ankle RoM and offloading the foot, with specific interest taken to lower extremity joint kinetics, kinematics, foot pressure, and spatiotemporal parameters of gait. The studies included in this review were not without methodological limitations; there were no longitudinal studies retrieved by this review and all included studies used healthy participants, so findings might not be transferrable to patients with pathologies that commonly receive CAM boot prescription. Furthermore, the studies in this review used a variety of different CAM boots which vary in design. Therefore, any inconsistencies in findings could be due to variability in performance between CAM boot designs and findings should not be generalised across different designs. These methodological inconsistencies compromise any between‐study comparisons and any such comparison should be interpreted with some caution.

**FIGURE 2:**
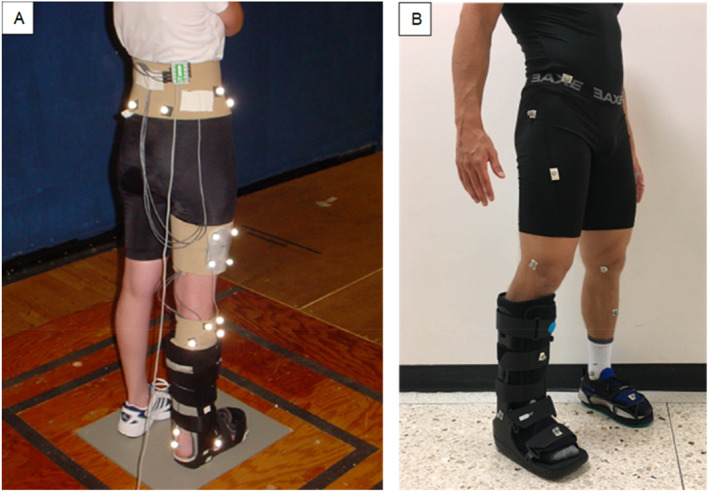
(A) The boot and marker set used by Zhang et al. (10), and (B) the boot and marker set used by Bruening et al. (7).

This systematic review suggests that CAM boots can be used to restrict ankle RoM [1, 5, 26], although the findings of Zhang et al. [24] opposed this. Whilst their method allows for in‐boot analysis of the movement of the foot and ankle, it potentially compromised the structural integrity of the boot removing substantial parts of the CAM boot, as can be seen in Figure 2. It should be considered, in future research, that cut outs of this size which expose large portions of the foot and ankle could have serious consequences on the structural integrity of the boots and reduce the validity of results, with the potential for variables such as ankle RoM to be inaccurate. This can be supported with a comparison to studies which adopt different analysis methods. Methods placing retroreflective markers on the outside of the boot where it is thought that the anatomical landmarks might be (Figure 2) do not have any consequences regarding the structural integrity of the boots. However, it could be suggested that “real” ankle RoM is not being measured, but instead, the movement of the boot is captured. Nonetheless, studies using this methodological approach contradict the findings of Zhang et al. [24], suggesting that ankle RoM is reduced and the ankle joint is immobilised by CAM boots, with 13.2° RoM in the CAM boot compared to 48.6° in the shoe condition [5]. McHenry et al. [1] and Nahm et al. [26] used fluoroscopic imaging to non‐invasively analyze in‐boot ankle joint kinematics, having no structural impact on the CAM boot yet allowing “actual” ankle movement to be measured. Both studies showed significant reductions in ankle RoM when tall CAM boots were compared to the respective control groups, suggesting that tall CAM boots can effectively restrict the ankle. McHenry et al. [1] also found short CAM boots significantly reduce ankle RoM compared to barefoot walking (control), whereas Nahm et al. [26] did not when comparing short CAM boots to shoe walking (control) during weightbearing. It should be noted that these authors took a different approach to measure the sagittal tibiotalar RoM, Nahm et al. [26] measured RoM using three defined points in the stance phase (heel strike, foot flat, and toe‐off), whereas McHenry et al. [1] used the difference between peak plantarflexion and dorsiflexion. This might explain the difference in their control groups with ankle RoM and subsequent lack of significant difference between control and short CAM boots RoM reported by Nahm et al. [26]. Nonetheless, both studies still found significance between boot differences. This suggests that tall CAM boots are most effective for restricting ankle RoM. This is further supported by the significant difference shown by McHenry et al. [1] and Nahm et al. [26] when comparing tall CAM boots to short CAM boots (*p* ≤ 0.05 and *p* = 0.002, respectively).

This narrative synthesis has identified that joint kinetics and kinematics at the neighboring joints (i.e., hip and knee) are altered which might give reason to the common occurrence of secondary site pain. Ipsilateral knee and contralateral hip pain are two of the most frequently reported sites of pain during and following CAM boot wear [2]. It could be suggested that this is because of the increased work done by these joints [5]. However, research to support this, or suggest the contrary, is limited. Increased ipsilateral knee flexion [6, 20, 24] with the additional mass of a CAM boot could contribute to this also being a frequently reported site of pain. The increased knee flexion poses issues to the iliotibial band as increased flexion causes friction with the lateral femoral epicondyle, causing inflammation [30]. Different boot designs can impose different alterations to gait biomechanics [6, 29]. Sommer et al. [29] showed knee kinematics to be significantly different between CAM boot designs with a VACOped boot having a lower knee RoM than Künzli and Orthotech boots. Similarly, knee and hip kinetics also differ depending on CAM boot design, with significant differences in joint moments [6, 29].

The findings of this systematic review illustrate that CAM boots can be used to unload pressure from the forefoot but not the hindfoot, as all but one of the included studies in this review found a posterior shift in foot pressure [23]. The degree to which the forefoot is offloaded can be amended by altering the degree of ankle plantarflexion within the CAM boot [27], with a significant correlation found between a reduction in forefoot pressure and the degree of plantarflexion [25]. However, if the primary goal is to offload the forefoot, it could be suggested that a tall CAM boot is not the most calculated approach. Short and tall CAM boots [26] are both shown to significantly reduce peak plantar pressures, but short boots are not as restrictive to ankle RoM [26] and could therefore reduce the risk of secondary site joint pain and muscle atrophy of the gastrocnemius and soleus through disuse [25], thus helping to avoiding potential long‐term muscle structural and architectural deficits. Furthermore, short and tall CAM boots significantly decrease relative forefoot pressures (*p* < 0.001) [28] which could advise their use for pathologies where regional offloading is required, such as diabetic patients for the treatment of plantar ulcerations. The influence of short CAM boot offloading whilst maintain more ankle RoM than tall CAM boots might make them better aligned for treating such pathologies with this greater range of ankle RoM potentially maintaining a more natural gait pattern and reducing secondary site injury and pain incidence.

### 4.1 Strengths and limitations

To our knowledge, this is the first systematic review evaluating the effectiveness of CAM boots in limiting ankle kinetics, kinematics, and plantar pressure. Extensive searches, with no restrictions on study type besides reviews, was undertaken to identify all studies published to date on this topic. However, we do acknowledge that we excluded pathological cohorts. Instead, healthy participants' data were reported to understand the biomechanical effects of CAM boots on the lower limb, excluding cofounders that specific pathology might have on lower limb kinetics and kinematics. Therefore, the application to a clinical population may be limited, but the findings of this review do provide insight to the mechanisms by which the use of CAM boots can impact function. A meta‐analysis was not performed because of the fundamental and methodological differences between papers. However, a narrative synthesis provides an in‐depth overview of the effects of CAM boot use that will help inform clinicians of the potential benefits and secondary implications of CAM boot prescription in clinical practice. The construction of the search strategy ensured a full and comprehensive search was conducted, but we accept that some specific terms might have been missed, so the inclusion of a hand search was necessary in the search process because of the unusual nature of terminology around CAM boots and biomechanics, ensuring all relevant research was included.

## 5 CONCLUSION

In summary, this systematic review shows that CAM boots restrict ankle RoM, although there is a lack of research directly quantifying in‐boot kinematics of the ankle joint during CAM boot wear without the risk of impacting structural integrity. It can also be concluded that CAM boots are an effective measure for offloading the forefoot, although short CAM boots are a more appropriate orthotic where forefoot offloading is the primary or sole purpose. This systematic review has highlighted that future research should seek to measure in‐boot kinematics without substantially affecting the structural integrity of the CAM boot. This approach would permit a more natural gait pattern and a higher number of trials that can be collected in one session than previously used methods of video fluoroscopy. The studies included in this review used only healthy populations, limiting the applicability of such studies to a clinical environment and pathological population. Longitudinal studies analyzing the effects of CAM boot wear in patients living with specific pathologies are required to fully understand the effectiveness of CAM boots for rehabilitation.

## AUTHOR CONTRIBUTIONS

**Mason L. Stolycia**: Conceptualization (equal); writing – original draft (lead); writing – review and editing (equal); formal analysis (lead); investigation (lead). **David E. Lunn**: Conceptualization (equal); writing – review and editing (equal); methodology (equal); project administration (co‐lead). **Josh Walker**: Conceptualization (equal); writing – review and editing (equal); methodology (equal); project administration (co‐lead). **Will Stanier**: Investigation (contribution). **Richard A. Wilkins**: Conceptualization (equal); writing – review and editing (equal); formal analysis (contribution).

## CONFLICT OF INTEREST STATEMENT

All authors declare no conflicts of interest.

## ETHICS STATEMENT

No IRB or ethical approval was needed due to the nature of this systematic review.

## Supporting information

Supporting Information S1

## References

[1] McHenry, B. D. , E. L. Exten , J. A. Cross , K. M. Kruger , B. Law , J. M. Fritz , and Gerald Harris . 2017. “Sagittal Subtalar and Talocrural Joint Assessment during Ambulation with Controlled Ankle Movement (CAM) Boots.” Foot & Ankle International 38(11): 1260–1266. 10.1177/1071100717723129. 28800714

[2] Ready, L. V. , E. G. Fisk , W. Ciurylo , C. P. Chiodo , E. M. Bluman , and J. T. Smith . 2018. “Associated Joint Pain with Controlled Ankle Movement Walker Boot Wear.” J Am Acad Orthop Surg Glob Res Rev 2(12): e044. 10.5435/jaaosglobal-d-18-00044. 30680366 PMC6336574

[3] NICE . Recommendations | Diabetic Foot Problems: Prevention and Management | Guidance. NICE 2015 [Available from: https://www.nice.org.uk/guidance/ng19/chapter/Recommendations#diabetic‐foot‐ulcer

[4] Aujla, R. S. , S. Patel , A. Jones , and M. Bhatia . 2019. “Non‐operative Functional Treatment for Acute Achilles Tendon Ruptures: The Leicester Achilles Management Protocol (LAMP).” Injury 50(4): 995–999. 10.1016/j.injury.2019.03.007. 30898390

[5] Bruening, D. A. , S. C. Huber , D. J. Parry , A. R. Hillier , A. E. M. Hayward , and J. K. Grover . 2022. “The Effect of Existing and Novel Walker Boot Designs on Offloading and Gait Mechanics.” Medical Engineering & Physics 108: 103890. 10.1016/j.medengphy.2022.103890. 36195362

[6] Richards, J. , K. Payne , D. Myatt , and A. Chohan . 2016. “Do Orthotic Walkers Affect Knee and Hip Function during Gait?” Prosthetics and Orthotics International 40(1): 137–141. 10.1177/0309364614546525. 25239143

[7] Golightly, Y. M. , K. D. Allen , C. G. Helmick , J. B. Renner , and J. M. Jordan . 2009. “Symptoms of the Knee and Hip in Individuals with and without Limb Length Inequality.” Osteoarthritis and Cartilage 17(5): 596–600. 10.1016/j.joca.2008.11.005. 19095470 PMC4082183

[8] Craik, J. D. , A. Clark , J. Hendry , A. H. Sott , and P. D. Hamilton . 2015. “The Effect of Ankle Joint Immobilization on Lower Limb Venous Flow.” Foot & Ankle International 36(1): 18–23. 10.1177/1071100714552823. 25249319

[9] Ho, E. , and A. Omari . 2017. “Prevalence of Acute Deep Vein Thrombosis in Patients with Ankle and Foot Fractures Treated with Nonoperative Management‐A Pilot Study.” International Journal of Angiology 26(1): 53–59. 10.1055/s-0035-1556054. PMC5330759 28255217

[10] Smyth, N. A. , P. Abbasi , C. D. C. Netto , S. M. Michnick , N. D. Casscells , B. G. Parks , and Lew C. Schon . 2019. “The Effect of Immobilization Devices on Contact Pressures of the Ankle and Hindfoot.” Foot Ankle Orthop 4(4): 2473011419S0040. 10.1177/2473011419s00401.

[11] Beck, J. J. , V. Kang , A. Bennett , S. Bloom , and N. J. Jackson . 2023. “Controlled Ankle Movement (CAM) Boot Provides Improved Outcomes with Lower Complications Than Short Leg Walking Cast in Low‐Energy Pediatric Lateral Ankle Injuries: A Prospective, Randomized Study.” Journal of Pediatric Orthopaedics 43(7): 418–423. 10.1097/bpo.0000000000002425. 37130811

[12] Abbott, A. , M. Bird , S. M. Brown , E. Wild , G. Stewart , and M. K. Mulcahey . 2020. “Part II: Presentation, Diagnosis, Classification, Treatment, and Prevention of Stress Fractures in Female Athletes.” The Physician and Sportsmedicine 48(1): 25–32. 10.1080/00913847.2019.1636546. 31295036

[13] Bradman, K. , K. Stannage , S. O'Brien , S. Green , N. Bear , and M. Borland . 2021. “Randomised Controlled Trial Comparing Immobilisation in Above‐Knee Plaster of Paris to Controlled Ankle Motion Boots in Undisplaced Paediatric Spiral Tibial Fractures.” Emergency Medicine Journal 38(8): 600–606. 10.1136/emermed-2020-210299. 34158387

[14] Firoozabadi, R. , E. Harnden , and J. C. Krieg . 2015. “Immediate Weight‐Bearing after Ankle Fracture Fixation.” Adv Orthop 2015: 491976ss. 10.1155/2015/491976. PMC4345246 25785201

[15] Hutchison, A. M. , C. Topliss , D. Beard , R. M. Evans , and P. Williams . 2015. “The Treatment of a Rupture of the Achilles Tendon Using a Dedicated Management Programme.” Bone & Joint J 97‐b(4): 510–515. 10.1302/0301-620x.97b4.35314. 25820890

[16] Walters, E. T. , and P. J. Kim . 2018. “Diabetic Foot Ulcer: Prevention, Management, and Controversies.” Current Trauma Reports 4: 273–283. 10.1007/s40719-018-0151-1.

[17] Harvey, W. F. , M. Yang , T. D. Cooke , N. A. Segal , N. Lane , C. E. Lewis , and D. T. Felson . 2010. “Association of Leg‐Length Inequality with Knee Osteoarthritis: a Cohort Study.” Annals of Internal Medicine 152(5): 287–295. 10.7326/0003-4819-152-5-201003020-00006. 20194234 PMC2909027

[18] Browne, M. G. , and J. R. Franz . 2017. “The Independent Effects of Speed and Propulsive Force on Joint Power Generation in Walking.” Journal of Biomechanics 55: 48–55. 10.1016/j.jbiomech.2017.02.011. 28262285 PMC5555113

[19] Hunt, K. J. , Y. Goeb , R. Esparza , M. Malone , R. Shultz , and G. Matheson . 2014. “Site‐specific Loading at the Fifth Metatarsal Base in Rehabilitative Devices: Implications for Jones Fracture Treatment.” PM & R: The Journal of Injury, Function, and Rehabilitation 6(11): 1022–1029. 10.1016/j.pmrj.2014.05.011. 24880059

[20] Gulgin, H. , K. Hall , A. Luzadre , and E. Kayfish . 2018. “3D Gait Analysis with and without an Orthopedic Walking Boot.” Gait & Posture 59: 76–82. 10.1016/j.gaitpost.2017.09.024. 29020659

[21] Page, M. J. , J. E. McKenzie , P. M. Bossuyt , I. Boutron , T. C. Hoffmann , C. D. Mulrow , Larissa Shamseer , et al. 2021. “The PRISMA 2020 Statement: An Updated Guideline for Reporting Systematic Reviews.” BMJ 372: n71. 10.1136/bmj.n71. 33782057 PMC8005924

[22] Pollo, F. E. , T. L. Gowling , and R. W. Jackson . 1999. “Walking Boot Design: A Gait Analysis Study.” Orthopedics 22(5): 503–507. 10348111

[23] Lawless, M. W. , G. T. Reveal , and R. T. Laughlin . 2001. “Foot Pressures during Gait: A Comparison of Techniques for Reducing Pressure Points.” Foot & Ankle International 22(7): 594–597. 11503987 10.1177/107110070102200712

[24] Zhang, S. , K. G. Clowers , and D. Powell . 2006. “Ground Reaction Force and 3D Biomechanical Characteristics of Walking in Short‐Leg Walkers.” Gait & Posture 24(4): 487–492. 16414263 10.1016/j.gaitpost.2005.12.003

[25] Kearney, R. S. , S. E. Lamb , J. Achten , N. R. Parsons , and M. L. Costa . 2011. “In‐Shoe Plantar Pressures within Ankle‐Foot Orthoses: Implications for the Management of Achilles Tendon Ruptures.” The American Journal of Sports Medicine 39(12): 2679–2685. 21908718 10.1177/0363546511420809

[26] Nahm, N. , M. J. Bey , S. Liu , and S. T. Guthrie . 2019. “Ankle Motion and Offloading in Short Leg Cast and Low and High Fracture Boots.” Foot & Ankle International 40(12): 1416–1423. 31423825 10.1177/1071100719868721

[27] Fröberg, Å. , M. Mårtensson , and A. Arndt . 2020. “The Effect of Ankle Foot Orthosis' Design and Degree of Dorsiflexion on Achilles Tendon Biomechanics‐Tendon Displacement, Lower Leg Muscle Activation, and Plantar Pressure during Walking.” Frontiers in sports and active living 2: 16. 33345010 10.3389/fspor.2020.00016 PMC7739684

[28] Ehrnthaller, C. , K. Rellensmann , S. F. Baumbach , M. Wuehr , R. Schniepp , M. M. Saller , W. Böcker , and H. Polzer . 2022. Pedobarographic Evaluation of Five Commonly Used Orthoses for the Lower Extremity. Archives of Orthopaedic and Trauma Surgery. 10.1007/s00402-022-04729-2 PMC10293377 36571629

[29] Sommer, B. , A. Hollenstein , and E. Graf . 2022. “Stability Boots for the Treatment of Achilles Tendon Injuries: Gait Analysis of Healthy Participants.” Gait & Posture 91: 131–136. 34689070 10.1016/j.gaitpost.2021.10.009

[30] Patel, D. R. , and A. Villalobos . 2017. “Evaluation and Management of Knee Pain in Young Athletes: Overuse Injuries of the Knee.” Translational Pediatrics 6(3): 190–198. 10.21037/tp.2017.04.05. 28795010 PMC5532199

