# Supplementary material for: Biomechanical effectiveness of controlled ankle motion boots: A systematic review and narrative synthesis
Source: J Foot Ankle Res. 2024 Jul 17;17(3):e12044. doi: 10.1002/jfa2.12044 (PMC11633339; doi:10.1002/jfa2.12044)
Supplement: Supplementary file 1 — Supporting Information S1 [file JFA2-17-e12044-s001.docx]

**Systematic Review Search Terms**

**EBSCOhost**

1. “cam” OR “controlled ankle motion” OR “controlled ankle movement”
2. “range of motion” OR “rom” OR “range of movement” OR “hinge*”
3. “stability”
4. “protect” OR “protection”
5. “Ossur”
6. “Ottobock”
7. “Medi”
8. “Oped”
9. “Enovis”
10. “boot*” OR “walker*” OR “shoe*” OR “orthoses” OR “orthotic*” OR “orthosis” OR “brace*” OR “cast*”
11. 1 OR 2 OR 3 OR 4 OR 5 OR 6 OR 7 OR 8 OR 9
12. 10 AND 11 OR “walker*”
13. “fracture*”
14. “diabetes”
15. “Achilles”
16. 13 OR 14 OR 15
17. “offload*”
18. “pressure”
19. “kinetic*”
20. “kinematic*”
21. “biomechanics” OR “biomechanical”
22. 17 OR 18 OR 19 OR 20 or 21
23. “foot”
24. “ankle”
25. 23 OR 24
26. 12 AND 16 AND 22 AND 25

**PUBMED**

("cam"[Title/Abstract] OR "controlled ankle motion"[Title/Abstract] OR "controlled ankle movement"[Title/Abstract] OR ("range of motion"[Title/Abstract] OR "rom"[Title/Abstract] OR "range of movement"[Title/Abstract]) OR "stability"[Title/Abstract] OR ("protect"[Title/Abstract] OR "protection"[Title/Abstract]) OR "ossur"[Title/Abstract] OR "ottobock"[Title/Abstract] OR "medi"[Title/Abstract] OR "oped"[Title/Abstract] OR "walker*"[Title/Abstract]) AND ("boot*"[Title/Abstract] OR "walker*"[Title/Abstract] OR "shoe*"[Title/Abstract] OR "orthoses"[Title/Abstract] OR "orthotic*"[Title/Abstract] OR "orthosis"[Title/Abstract] OR "brace*"[Title/Abstract] OR "cast*"[Title/Abstract]) AND ("fracture*"[Title/Abstract] OR "diabetes"[Title/Abstract] OR "achilles"[Title/Abstract]) AND ("offload*"[Title/Abstract] OR "pressure"[Title/Abstract] OR "kinetic*"[Title/Abstract] OR "kinematic*"[Title/Abstract] OR ("biomechanics"[Title/Abstract] OR "biomechanical"[Title/Abstract])) AND ("foot"[Title/Abstract] OR "ankle"[Title/Abstract])
